# Laser Pulse-Driven Multi-Sensor Time Synchronization Method for LiDAR Systems

**DOI:** 10.3390/s25247555

**Published:** 2025-12-12

**Authors:** Jiazhi Yang, Xingguo Han, Wenzhong Deng, Hong Jin, Biao Zhang

**Affiliations:** 1 Guangxi Region Precious Metal Materials Advanced Process Research Center, Guilin University of Aerospace Technology, Guilin 541004, China; jiazhi.yang@guat.edu.cn (J.Y.);; 2 University Engineering Research Center of Non-Standard Intelligent Equipment and Process Control Technology, Guilin University of Aerospace Technology, Guilin 541004, China; 3 Guangxi Key Laboratory of Special Engineering Equipment and Control, Guilin University of Aerospace Technology, Guilin 541004, China; 4 School of Computer Science and Engineerin, Guilin University of Technology, Guilin 541004, China

**Keywords:** LiDAR, time synchronization, multi-sensor, laser pulse driven, MCU

## Abstract

Multi-sensor systems require precise time synchronization for accurate data fusion. However, currently prevalent software time synchronization methods often rely on clocks provided by the Global Navigation Satellite System (GNSS), which may not offer high accuracy and can be easily affected by issues with GNSS signals. To address this limitation, this study introduces a novel laser pulse-driven time synchronization (LPTS) method in our custom-developed Light Detecting and Ranging (LiDAR) system. The LPTS method uses electrical pulses, synchronized with laser beams as the time synchronization source, driving the Micro-Controller Unit (MCU) timer within the control system to count with a timing accuracy of 0.1 μs and to timestamp the data from the Positioning and Orientation System (POS) unit or laser scanner unit. By employing interpolation techniques, the POS and laser scanner data are precisely synchronized with laser pulses, ensuring strict correlation through their timestamps. In this article, the working principles and experimental methods of both traditional time synchronization (TRTS) and LPTS methods are discussed. We have implemented both methods on experimental platforms, and the results demonstrate that the LPTS method circumvents the dependency on external time references for inter-sensor alignment and minimizes the impact of laser jitter stemming from third-party time references, without requiring additional hardware. Moreover, it elevates the internal time synchronization resolution to 0.1 μs and significantly improves relative timing precision.

## 1. Introduction

A multi-sensor system refers to a system that obtains and combines data from multiple sensors, with data fusion being a pivotal aspect in such systems. In the context of Simultaneous Localization and Mapping (SLAM) systems, multi-sensor fusion is a critical issue, as it involves integrating data from different sensors to enhance the robustness, accuracy, and reliability of the system [1,2,3]. However, the sampling frequencies and timestamps of different sensors may vary, leading to data asynchronicity. Hence, a high-precision time synchronization mechanism is required to align data temporally [4,5].

Light Detecting and Ranging (LiDAR), as an important SLAM method that uses laser beams to measure distance, velocity, and other properties, has found widespread applications in various fields including autonomous driving [6,7], environmental monitoring [8], geological exploration [9], and robot navigation [10,11,12]. However, LiDAR shares the same multi-sensor data fusion challenges as other sensing technologies. A typical LiDAR system consists of multiple sensor units that output data at different frequencies, including laser trigger pulses, positioning and attitude data from the Positioning and Orientation System (POS), rotation angles, the number of revolutions, and the rotational speed of the laser scanner. These data require precise time synchronization during the generation of LiDAR point cloud data. The accuracy and stability of internal multi-sensor time synchronization play a crucial role in ensuring data consistency, target tracking precision, and system efficiency improvement [13,14,15]. More precise time-synchronized multi-sensor data enhances the quality of inputs for sensor fusion machine learning (ML) models, thereby advancing the performance of artificial intelligence (AI) applications [16,17].

There are many factors that affect the accuracy of multi-sensor time synchronization, namely, time reference error, signal transmission delay, system response error, laser jitter error, and timestamping error. The former four errors are tied to hardware or system design. For instance, the reference error refers to the inherent error in the pulse signal that serves as the benchmark. If the benchmark experiences jitter or loss, it can directly affect the accuracy of time synchronization. System transmission error refers to the unexpected delays or loss of synchronization data between data acquisition time and synchronization time, caused by interference or inherent issues in external data acquisition devices such as POS, which lead to data transmission delays or data loss. System response error refers to the delay error that occurs during the acquisition, time synchronization, and storage of external data due to insufficient real-time performance of the Micro-Controller Unit (MCU) within the control system. Laser jitter refers to the temporal fluctuation between the laser trigger signal and the actual emission of the laser beam, which is inherent to the characteristics of the laser device. If the laser trigger signal is used as the synchronization signal for the laser echo, it will inevitably introduce laser jitter error [18,19].

In addition to the previously mentioned factors influencing the time synchronization precision of multi-sensor systems, the operational and environmental conditions of the system itself also have an impact on multi-sensor time synchronization. Such factors include conditions of the workplace, carrier platform vibrations and shocks, electromagnetic environment, and lighting conditions. More specifically, positioning and precise timing services cannot be provided in Global Navigation Satellite System (GNSS)-denied environments such as indoors and caves. Variations in temperature and humidity have the potential to induce drifts in sensor operating characteristics, particularly in clock circuits where temperature-induced drift effects may occur. Vibrations and shocks from the carrier platform may disrupt the operation of the POS system, thereby compromising the accuracy of its positioning and attitude measurements. Exposure of the multi-sensor system to intense electromagnetic interference can negatively impact internal circuitry, leading to noise generation or even system malfunction. Furthermore, excessively intense ambient light has the capacity to disrupt the photoelectric system, possibly resulting in the blinding of photoelectric devices.

Timestamping error is linked to the selection of time synchronization methods and software algorithms. Implementing an effective time synchronization principle along with suitable software algorithms can significantly minimize timestamping error. Common methods for internal multi-sensor time synchronization in LiDAR systems include the following categories, and their pros and cons are listed in Table 1.

(1) Hardware synchronization method: This method employs hardware circuits [14,20], Field-Programmable Gate Arrays (FPGAs) [21], and other kinds of specified hardware [22,23] to synchronize the clocks of multiple sensors, ensuring that all sensors share the same time reference. This approach is direct and simple, avoiding the need for additional communication and protocols, resulting in lower latency. However, it requires specialized hardware design and implementation, which may increase costs and complexities.

(2) Software synchronization method: This method utilizes software algorithms to synchronize the clocks of multiple sensors. It can be implemented through the Precision Time Protocol (PTP) [24], or other custom protocols [25,26]. This approach is flexible and does not require additional hardware, but it may introduce some delays and complexities in communication and protocol.

In recent years, within the SLAM systems for robotics or autonomous driving, there has been a surge of research adopting continuous-time trajectory estimation [27,28], extended Kalman filters [29], or deep-learning-based frameworks [30] to achieve LiDAR–Inertial Measurement Unit (IMU)–Camera time synchronization. Although these methods provide high synchronization accuracy, they require significant computational resources, making it challenging to achieve real-time online time synchronization in high-frequency, high-precision SLAM applications.

(3) Hybrid synchronization method: This method combines hardware and software synchronization techniques to achieve high-precision time synchronization. It can be implemented through a combination of GNSS [14] or other hardware circuits and software algorithms [4]. This approach can achieve high precision and low latency, but it requires additional hardware and complex software design.

The aforementioned time synchronization methods all use a precise third-party time reference, either internal or external traditional time synchronization (TRTS), to align various frequency data within the LiDAR system. This approach ensures high synchronization accuracy and minimizes timestamping errors. However, the following issues still exist: First, when assigning timestamps to LiDAR data from distinct sensors, a timestamping error of ±1 time stepping arises for each data. This error compounds to a timestamping error of ±2 time stepping among the different frequency data of the LiDAR during point cloud processing. Secondly, laser pulses exhibit time jitter, leading to discrepancies between the laser range data and interpolated data derived from other low-frequency sensors during point cloud generation.

To address the issues associated with external time synchronization methods like unreliable or missing external clocks, and internal hardware synchronization methods such as increased hardware costs and reduced reliability due to additional hardware components, we propose an internal time synchronization method, i.e., laser pulse-driven time synchronization (LPTS), in our custom-developed LiDAR system. To generate a time reference for other low-frequency data within the LiDAR system, electrical pulses synchronized with laser beams are utilized to drive the MCU’s high-precision internal timer. The LPTS method can decrease the timestamping error between the different frequency data of the LiDAR. Furthermore, the LPTS method adopts an electrical signal synchronized with the laser as the time synchronization reference signal, thereby circumventing the issue of laser timing jitter.

The results demonstrate that the LPTS method effectively reduces the impact of laser jitter and achieves better synchronization accuracy compared to the TRTS method, without requiring additional hardware such as an FPGA. Instead, it relies solely on the peripherals integrated within the MCU, thereby delivering enhanced performance at a lower hardware cost, making it well-suited for time synchronization in multi-sensor systems.

## 2. Timestamping Principles and Error Analysis

As discussed in the previous section, timestamping errors significantly impact the precise time synchronization in multi-sensor systems. It is crucial to distinguish between absolute time accuracy and relative synchronization precision. While GNSS provides atomic-level absolute time, the LPTS method prioritizes the relative temporal alignment between the laser emission and sensor data, thereby bypassing the jitter inherent in the laser’s response to external GNSS triggers. Traditional TRTS methods exhibit a ±2 times time stepping error, whereas the LPTS method reduces this to ±1. Additionally, the LPTS method effectively eliminates the influence of laser jitter. This section will delve into the principles and timestamping errors associated with both the TRTS and LPTS methods.

### 2.1. TRTS Method

**Figure 1:**
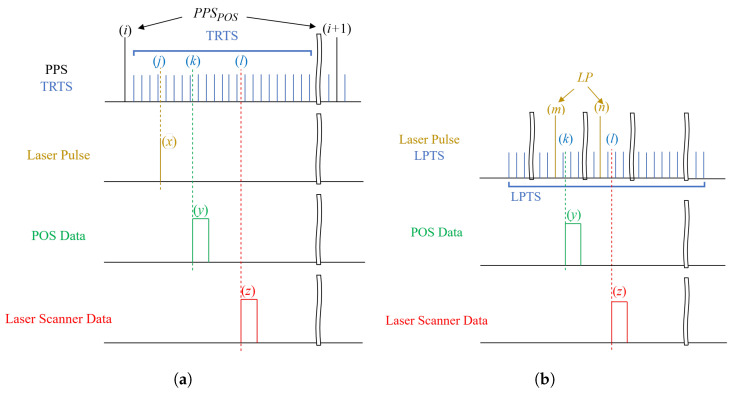
Schematic diagram of timestamping principles. (**a**) TRTS methods use PPS, while the (**b**) LPTS method uses laser pulse as time references to generate $10^{7}$ Hz timestamping pulses. The wavy markers indicate the omitted portions in the timelines, the dotted lines represent isochronous points on the timebase, while arrows point to distinct PPS or laser pulse signals. Letters *i*, *j*, *k*, and *l* denote the sequence numbers of the PPS signal, the first laser pulse, the first POS data, and the first laser scanner data after the *i*-th PPS signal, respectively. Natural numbers *x*, *y*, and *z* represent the sequence numbers of laser pulses, POS data, and laser scanner data between two consecutive PPS signals, respectively.

Most TRTS methods depend on either an external or internal time reference, often a Pulse Per Second (PPS) signal, to generate more precise time references with an accuracy of up to 1 μs [14]. These time references are subsequently utilized to timestamp the incoming sensor data of varying frequencies. The schematic diagram illustrating the timestamping principles of the TRTS methods is presented in Figure 1a.

To enable a comprehensive comparison between the TRTS and LPTS methods, we assume the following for the TRTS method illustrated in Figure 1a: the sequence number of the initial PPS pulse from the POS unit is denoted as $PPS_{POS}\left(i\right)$. Pulses generated at a frequency of $10^{7}$ Hz or higher are used as TRTS timestamps, corresponding to a time stepping of 0.1 μs. Furthermore, the timestamps corresponding to the first laser pulse, the first POS data, and the first laser scanner data following $PPS_{POS}\left(i\right)$ are TRTS(j), TRTS(k), and TRTS(l), respectively. It should be noted that the relative magnitudes of *j*, *k*, and *l* are random, and their temporal sequence in Figure 1a is for illustrative purposes only.

The timestamp of the *x*-th laser pulse, occurring between $PPS_{POS}\left(i\right)$ and $PPS_{POS}\left(i+1\right)$, can be expressed by (1), where *x* is a natural number greater than 0.

(1) $$\begin{array}{c} TS_{laser}^{TRTS}\left(x\right)=PPS_{POS}\left(i\right)+TRTS\left(\frac{f_{TRTS}}{f_{laser}}\left(x-1\right)+j\pm 1\right) \end{array}$$

In (1), $f_{TRTS}$ and $f_{laser}$ represent the frequencies of TRTS and laser pulses, respectively. Taking into account system response error and laser jitter, a ±1 time stepping error may arise during the timestamping process.

The *y*-th timestamp of the POS data and the *z*-th timestamp of the laser scanner data, both falling between $PPS_{POS}\left(i\right)$ and $PPS_{POS}\left(i+1\right)$, can be represented by (2) and (3), respectively. Here, *y* and *z* are natural numbers greater than 0.

(2) $$\begin{array}{c} TS_{POS}^{TRTS}\left(y\right)=PPS_{POS}\left(i\right)+TRTS\left(\frac{f_{TRTS}}{f_{POS}}\left(y-1\right)+k\pm 1\right) \end{array}$$

(3) $$\begin{array}{c} TS_{scanner}^{TRTS}\left(z\right)=PPS_{POS}\left(i\right)+TRTS\left(\frac{f_{TRTS}}{f_{scanner}}\left(z-1\right)+l\pm 1\right) \end{array}$$

In (2), $f_{POS}$ represents the frequency of POS data, while in (3), $f_{scanner}$ denotes the frequency of laser scanner data.

In order to generate point cloud data, an interpolation process will be carried out on the POS data and the laser scanner data to align them with the laser pulse frequency. Only when the TRTS timestamps of the interpolated POS or laser scanner data coincide with those of the laser pulse can they be fused to generate a point cloud.

Following interpolation, the new timestamp $TS_{POS}^{TRTS}\left(y^{\prime }\right)$ for the interpolated POS data, located between $TS_{POS}^{TRTS}\left(y\right)$ and $TS_{POS}^{TRTS}\left(y+1\right)$, is described by (4), where $y^{\prime }$ is a natural number greater than 0, representing the TRTS time step (i.e., 0.1 μs) in the temporal sequence shown in Figure 1a. The ±1 times time stepping error becomes negligible after the interpolation process.

(4) $$TS_{POS}^{TRTS}\left(y^{\prime }\right)=TS_{POS}^{TRTS}\left(y\right)+TRTS\left(y^{\prime }\right)$$

The new timestamp $TS_{scanner}^{TRTS}\left(z^{\prime }\right)$ for the interpolated laser scanner data, lying between $TS_{scanner}^{TRTS}\left(z\right)$ and $TS_{scanner}^{TRTS}\left(z+1\right)$, can be expressed by (5). Here, $z^{\prime }$ is a natural number greater than 0.

(5) $$TS_{scanner}^{TRTS}\left(z^{\prime }\right)=TS_{scanner}^{TRTS}\left(z\right)+TRTS\left(z^{\prime }\right)$$

To achieve precise alignment with the laser pulse and the interpolated POS data, disregarding any timestamping errors, $TS_{POS}^{TRTS}\left(y^{\prime }\right)$ should equal $TS_{laser}^{TRTS}\left(x\right)$. Consequently, the conditions outlined in (6) and (7) must be satisfied.

(6) $$y⩽\frac{f_{POS}}{f_{laser}}x+\frac{f_{POS}}{f_{TRTS}}\left(j-k\right)⩽y+1$$

(7) $$\frac{f_{TRTS}}{f_{POS}}\left(y-1\right)+y^{\prime }+k=\frac{f_{TRTS}}{f_{laser}}\left(x-1\right)+j$$

Equation (6) constrains the *x* value corresponding to the interpolated $y^{\prime }$ to lie within the same range as $y^{\prime }$ itself (between *y* and y+1), whereas (7) establishes the quantitative relationships between the interpolated $y^{\prime }$ and the variables *x* and *y*, as well as the initial occurrence positions *j* and *k*.

The interpolated laser scanner data are also subject to similar conditions. The timestamping error of the TRTS methods, denoted as $E_{POS}^{TRTS}$, can be determined by subtracting (4) from (1), given that the conditions specified in (6) and (7) are satisfied. The outcome is represented by (8).

(8) $$E_{POS}^{TRTS}=TS_{POS}^{TRTS}\left(y^{\prime }\right)-TS_{laser}^{TRTS}\left(x\right)=TRTS\left(\pm 2\right)$$

$E_{POS}^{TRTS}$ can also be calculated by subtracting $TS_{laser}^{TRTS}\left(x\right)$ from $TS_{scanner}^{TRTS}\left(z^{\prime }\right)$, yielding the same result as (8). $E_{POS}^{TRTS}$ is ±0.2 μs due to the ±0.1 μs time step of TRTS.

### 2.2. LPTS Method

In the LPTS method illustrated in Figure 1b, pulses are generated at the same frequency as in the TRTS methods by referencing laser pulses (i.e., $10^{7}$ Hz), serving as LPTS timestamps. We assume that the laser pulse is represented by LP(x). Additionally, LPTS(k) represents the timestamp linked to the POS data following LP(m), while the initial laser scanner data following LP(n) and LPTS(l) are denoted accordingly. The integers *m* and *n* are defined as $m=y\times f_{laser}÷f_{POS}$ and $n=z\times f_{laser}÷f_{scanner}$, respectively. It is important to note that the calculations for *m* and *n* employ downward rounding to integer values. The downward rounding of *m* and *n* implies that POS data *y* and laser scanner data *z* occurred after laser pulses LP(m) and LP(n), respectively, but before the subsequent pulses LP(m+1) and LP(n+1).

Since the laser pulses serve as the time references in the LPTS method, the *x*-th laser pulse can be denoted by (9), where *x* is a natural number greater than 0.

(9) $$TS_{laser}^{LPTS}\left(x\right)=LP\left(x\right)+LPTS\left(0\right)$$

As the frequencies of the POS data and the laser scanner data are significantly lower than that of the laser pulse, it is possible that no POS data or laser scanner data is received between LP(x) and LP(x+1). The timestamps for the *y*-th POS data and *z*-th laser scanner data following LP(x) are represented by (10) and (11), respectively, where both *y* and *z* are natural numbers greater than 0.

(10) $$TS_{POS}^{LPTS}\left(y\right)=LP\left(m\right)+LPTS\left(k\pm 1\right)$$

(11) $$TS_{scanner}^{LPTS}\left(z\right)=LP\left(n\right)+LPTS\left(l\pm 1\right)$$

To align with the laser pulse frequency during point cloud data processing, it is necessary to interpolate both POS data and laser scanner data to match the laser pulse frequency. Equation (12) represents the new timestamp, denoted as $TS_{POS}^{LPTS}\left(y^{\prime \prime }\right)$, which falls between $TS_{POS}^{LPTS}\left(y\right)$ and $TS_{POS}^{LPTS}\left(y+1\right)$. Here, $y^{\prime \prime }$ is a natural number greater than 0, representing the LPTS time step (i.e., 0.1 μs) in the temporal sequence shown in Figure 1b.

(12) $$TS_{POS}^{LPTS}\left(y^{\prime \prime }\right)=TS_{POS}^{LPTS}\left(y\right)+LPTS\left(y^{\prime \prime }\right)$$

The new interpolated timestamp, denoted as $TS_{scanner}^{LPTS}\left(z^{\prime \prime }\right)$, which falls between $TS_{scanner}^{LPTS}\left(z\right)$ and $TS_{scanner}^{LPTS}\left(z+1\right)$, is expressed by (13). Here, $z^{\prime \prime }$ represents a natural number greater than 0.

(13) $$TS_{scanner}^{LPTS}\left(z^{\prime \prime }\right)=TS_{scanner}^{LPTS}\left(z\right)+LPTS\left(z^{\prime \prime }\right)$$

Under ideal conditions, for precise alignment with the interpolated POS data and the laser pulse, it is necessary that $TS_{POS}^{LPTS}\left(y^{\prime \prime }\right)$ equals $TS_{laser}^{LPTS}\left(x\right)$, provided that the conditions stated in (14) and (15) are satisfied.

(14) $$y⩽\frac{f_{POS}}{f_{laser}}\left(x\right)+\frac{f_{POS}}{f_{LPTS}}k⩽y+1$$

(15) $$\frac{f_{LPTS}}{f_{POS}}\left(y\right)+y^{\prime \prime }+k=\frac{f_{LPTS}}{f_{laser}}\left(x\right)$$

In (14) and (15), $f_{LPTS}$ represents the frequency of LPTS pulses. The interpolated laser scanner data are also subject to similar conditions. The timestamping error $E_{POS}^{LPTS}$ of the LPTS method can be determined by subtracting (12) from (9), given that the conditions of Equations (14) and (15) are satisfied. The outcome is represented by (16).

(16) $$E_{POS}^{LPTS}=TS_{POS}^{LPTS}\left(y^{\prime \prime }\right)-TS_{laser}^{LPTS}\left(x\right)=LPTS\left(\pm 1\right)$$

Upon comparing (8) with (16), it becomes evident that the timestamping error incurred by the LPTS method is half that of the TRTS method, assuming both methods possess the same timestamping resolution.

## 3. Experimental Setup

To discuss the experimental setup for implementing our proposed LPTS time synchronization method, the hardware and software of our custom-developed LiDAR system will be introduced in this section.

### 3.1. System Hardware

**Figure 2:**
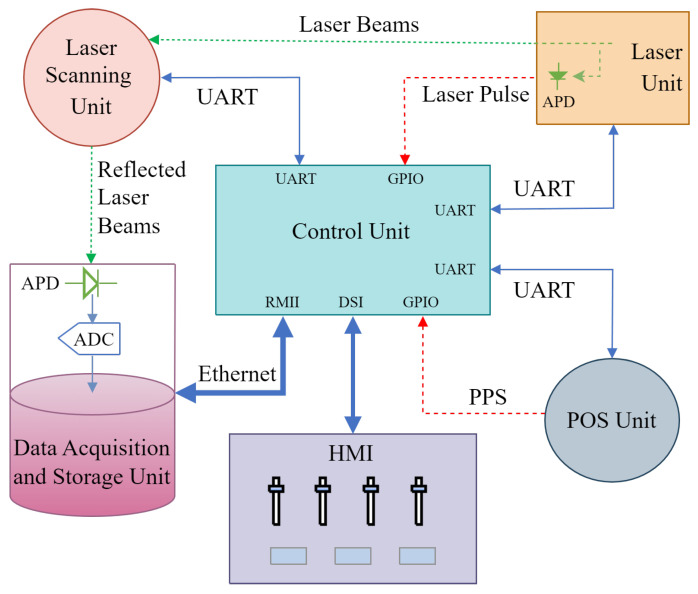
Block diagram of our custom-developed LiDAR system. The blue solid lines, red dashed lines, and green dotted lines signify the data connection channels, time synchronization connections, and laser beams among the units, respectively.

The block diagram for implementing the LPTS time synchronization method in our custom-developed LiDAR system is illustrated in Figure 2, comprising a control unit, laser unit, POS unit, laser scanner unit, data acquisition and storage unit, and Human–Machine Interface (HMI). The blue solid lines, red dashed lines, and green dotted lines signify the data connection channels, time synchronization connections, and laser beams among the units, respectively.

The core module of the LiDAR system is the control unit, which utilizes a high-performance MCU running an embedded real-time operating system to initialize, control start/stop functions, detect status, and manage errors of the system’s units. Data links are employed by the main control unit for communication and control with other units. The time synchronization signal within the LiDAR system is a Transistor–Transistor Logic (TTL) pulse signal synchronized with the laser beam from the laser unit. The control unit records and stores this synchronization pulse signal, which is then used to timestamp the scanning angle provided by the laser scanner unit and the positional, angular, and other data from the POS unit. The timestamped data frames are subsequently stored in the data acquisition and storage unit. After being collimated by the lens, the laser beam is directed by the laser scanner unit onto the target object. Subsequently, the reflected laser beam is once again directed by the laser scanner unit, focused by the lens, and finally illuminated onto the Avalanche Photo Diode (APD) within the data acquisition and storage unit, where it is converted into an electrical signal. The electrical signal is digitized and stored in the data acquisition and storage unit.

The key performances of the custom-developed LiDAR system are presented in Table 2.

#### 3.1.1. Control Unit

In our custom-developed LiDAR system, the control unit is tasked with initiating each unit, commencing and ceasing system operations, coordinating time synchronization, engaging with users, managing errors, and gathering data. Time synchronization plays a critical role as it impacts the integration of position, attitude, distance, angle, and other data produced by the LiDAR system, ultimately influencing the precision of SLAM.

Within the LiDAR system, the control unit is equipped with an STM32H743 MCU from STMicroelectronics, featuring a clock speed of 400 MHz, 2 MB of Flash memory, 1 MB of RAM, and 168 I/Os supporting up to 35 communication ports. This MCU is externally expanded with 32 MB of Quad Serial Peripheral Interface (QSPI) Flash and 32 MB of Synchronous Dynamic Random Access Memory (SDRAM), enhancing the control unit’s real-time performance and scalability in the LiDAR system. The embedded software operates on the FreeRTOS real-time operating system with a thread scheduling frequency of 1000 Hz and prioritizes interrupt responses to ensure timely system reactions. Furthermore, the embedded software leverages the TouchGFX HMI framework to deliver a seamless HMI and robust interaction capabilities, simplifying the configuration of LiDAR system parameters.

#### 3.1.2. Laser Unit

The laser unit comprises a semiconductor-pumped pulsed laser emitting at a wavelength of 532 nm, with a repetition frequency of 2 kHz, a pulse width of 3 ns, and a single pulse peak power of 200 kW. This laser can produce a TTL electrical pulsed signal in sync with the laser beam, serving as a reliable time reference for the LPTS method.

A lens is integrated at the outlet of the laser unit to collimate the emitted laser, ensuring that the spot diameter is less than 2 mm at a distance of 50 m.

The control unit interfaces with the laser unit via the Universal Asynchronous Receiver–Transmitter (UART) port, enabling functions such as laser initialization, parameter adjustment, control activation, status queries, and more.

#### 3.1.3. POS Unit

The POS unit utilizes the INS-DH-OEM by Inertial Labs, capable of reaching a maximum data update rate of 100 Hz via UART. It delivers horizontal and vertical positioning accuracies of 0.01 m and 0.02 m, respectively, in Real-Time Kinematic (RTK) mode. The unit provides a heading accuracy of 0.05 deg and a pitch and roll accuracy of 0.015 deg.

The HG4930 IMU built into the NS-DH-OEM has a vibration tolerance range within the frequency of 10–200 Hz, with a maximum vibration amplitude of 20 G (196 $m/s^{2}$).

#### 3.1.4. Laser Scanner Unit

The laser scanner unit employs a servo motor to maneuver a wedge reflector, with the servo motor attaining a maximum speed of 2000 rpm and offering a scanning angle accuracy of 0.072 deg. The servo motor driver connects to the control unit through an RS485 to the UART bus and communicates with the MCU through the Modbus protocol. The control unit can initiate the servo motor upon system startup, regulate its speed, and retrieve data on the motor’s angle and laps.

After being reflected and deflected by the laser scanner unit, the laser performs a circular scan on the target. The laser reflected back from the target is focused by the laser scanner unit and sent to the data acquisition and storage unit.

#### 3.1.5. Data Acquisition and Storage Unit

The LiDAR system captures and records the full waveform of the laser echo signal. The data acquisition and storage unit is equipped with two APD modules. These modules serve to convert the laser beam emitted by the laser device and the laser beam reflected from the target, respectively. During the subsequent point cloud data processing phase, the distance to the target is determined by calculating the time difference between the peaks of the two laser waveforms. Due to the considerable attenuation of the reflected laser beam and its potential coupling with noise, we have implemented filtering and amplification circuits preceding the APDs. This ensures that the amplitude of the converted electrical signal falls within the ±2.5 V range. Additionally, this unit incorporates four channels of 12-bit high-speed Analog-to-Digital Converters (ADCs), operating at a sampling rate of 2 Giga-Samples Per Second (GSPS), and offering 2 terabytes of data storage. The control unit configures the operational mode and parameters of the data acquisition and storage unit via Ethernet using the Reduced Media Independent Interface (RMII), transmitting timestamped valid data extracted from the POS unit, servo motor speed, rotation angle, and accumulated lap information to the storage unit at regular intervals. In the initial phase of LiDAR data post-processing, the full-waveform echo data is integrated with the information provided by the control unit while utilizing timestamp synchronization.

#### 3.1.6. UAV Platform

The unmanned aerial vehicle (UAV) platform used in the experiment is a hexacopter with a payload capacity of 25 kg, capable of carrying a LiDAR system weighing 18 kg. The battery pack carried by the unmanned aerial vehicle (UAV) can sustain a flight time of 2 h and provide a maximum power output of 500 W, fully meeting the 200 W power demand of the LiDAR system. Furthermore, the UAV can withstand winds of up to force three (speeds < 6 m/s). Flexible materials are used for connecting when installing the LiDAR system onto the UAV, aiming to reduce vibrations and thus minimize the impact on the operation of the IMU in the POS system.

The LiDAR system operates within a temperature range of 0–40 °C and functions optimally during the daytime, provided there is no direct sunlight exposure to the lens, as well as at night. The flight parameters adopted in the experiment are listed in Table 2.

After selecting and designing the various hardware components of the LiDAR system, it is necessary to consider the system’s requirements for shock and vibration resistance when operating on a UAV platform. When designing and assembling the various units of the LiDAR system, fixed connections should be utilized to ensure a consistent working environment for all components within the LiDAR system. At the same time, to guarantee the reliability of electronic components on the Printed Circuit Board (PCB), it is imperative to prioritize welding quality and spray a three-proof paint to fulfil insulation, moisture resistance, and corrosion resistance requirements.

**Figure 3:**
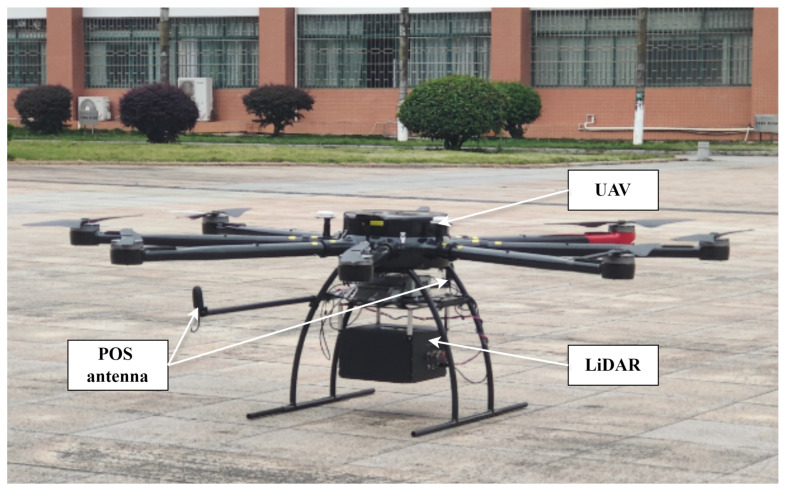
Experimental setup of our custom-developed LiDAR system.

The experimental setup of our custom-developed LiDAR system is shown in Figure 3. The LiDAR system is mounted on a UAV platform, and the laser beam is directed towards the target object. The POS unit is assembled on the UAV platform to provide real-time positioning and attitude data.

### 3.2. System Software

When designing system software, the primary considerations are software robustness and real-time responsiveness. Therefore, we chose a Real-Time Operating System (RTOS) and related software frameworks for the software design. Tasks with low real-time requirements are handled through task switching; tasks with high real-time requirements are responded through interrupts. Simultaneously, we adopted a mature Graphical User Interface (GUI) framework to design the HMI. These measures enable us to successfully integrate the LPTS method proposed in this paper into the LiDAR system, achieving high-precision time synchronization for multiple sensors.

**Figure 4:**
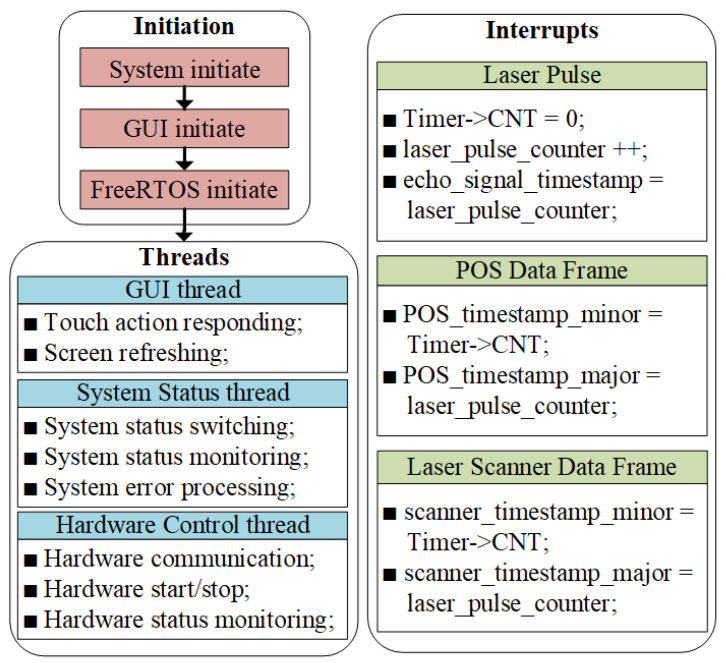
Software flowchart with the implementation of LPTS method in our custom-developed LiDAR system.

The software flowchart of the LiDAR system, with the LPTS method integrated, is described in Figure 4. Upon powering on the LiDAR system, the MCU within the control unit initiates the execution of specialized code. The initial step entails the control unit initializing various components, such as MCU peripherals, external units, GUI, and FreeRTOS. Upon completion of the initialization process, the entire LiDAR system is ready, triggering the start of thread scheduling by the RTOS.

Furthermore, the RTOS switches between threads, specifically the GUI thread, system status thread, and hardware control thread, in accordance with their respective priorities. The GUI thread is responsible for handling touch interactions and screen updates, and it facilitates an HMI. Simultaneously, the system status thread assesses system conditions, monitors errors, and administers error processing. The hardware control thread, on the other hand, manages communication with external hardware components including the POS unit, laser unit, laser scanner unit, and data acquisition and storage unit. This thread controls start/stop operations and monitors the status of these components.

Finally, to enhance the real-time performance of the LiDAR system, interrupts are utilized to respond to critical events like laser pulse counting, POS data frame timestamp, and laser scanner data frame timestamp. The system prioritizes interrupt responses to ensure timely system reactions. Specifically, the laser pulse capture interrupt is assigned the highest priority level in the Nested Vector Interrupt Controller (NVIC) to guarantee zero data loss during high-load operations.

A global variable, laser_pulse_counter, which tracks and stores the number of laser pulses, is the *x* in (9). Upon receiving a TTL pulse from the laser unit, the MCU triggers an interrupt, initiating the laser pulse interrupt service program. The timer counting register dedicated to precise time synchronization is reset, followed by incrementing the global variable laser_pulse_counter by 1. Additionally, another global variable, echo_signal_timestamp, representing the timestamp of the full-wave echo signal, synchronizes with the laser_pulse_counter. Subsequently, when data frames from the POS unit or the laser scanner unit are transmitted to the MCU, the corresponding interrupt is triggered. The timestamping process updates the POS_timestamp_major and scanner_timestamp_major variables with the contemporaneous value of the laser_pulse_counter, which are *m* and *n* in (10) and (11), respectively. The POS_timestamp_minor and scanner_timestamp_minor variables are refreshed based on the present value of the timer’s counting register, which represent the k±1 and l±1 in Equations (10) and 11, respectively.

Given the recorded parameters *x*, *m*, *n*, k±1, and l±1, the timestamps of the POS and laser scanner data can be calculated using the LPTS method through (9)–(11). Additionally, the interpolated timestamp of the POS data, aligned with the laser pulse, can be determined using (12). Similarly, the interpolated timestamp of the laser scanner data can be obtained through a comparable process.

## 4. Experimental Results and Analysis

Based on the theoretical analysis in Section 2, the LPTS method offers superior timestamping accuracy compared to traditional TRTS methods. To quantitatively evaluate this improvement, we conducted experiments using our custom-developed LiDAR system described in Section 3.

### 4.1. Implementation of TRTS and LPTS for Timestamping Error Evaluation

Traditional TRTS methods, specifically those relying on GNSS-derived PPS signals, depend on an external time reference to generate timestamps for the laser scanner data and POS data, often resulting in multi-stage error propagation due to signal transmission delays.

**Figure 5:**
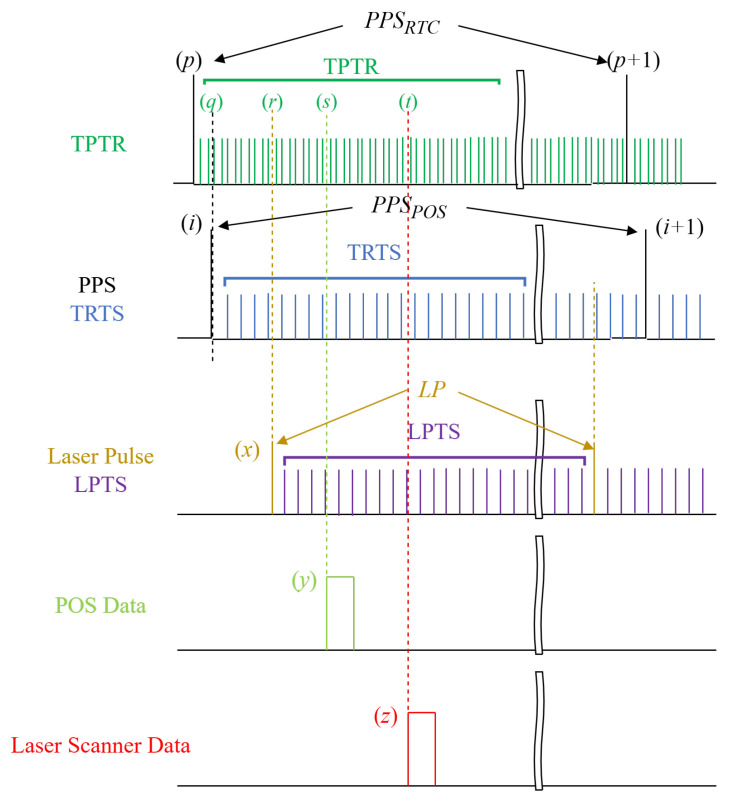
Schematic diagram of evaluating the relative timestamping errors of TRTS and LPTS methods by the TPTR method. The wavy markers indicate the omitted portions in the timelines, the dotted lines represent isochronous points on the timebase, while arrows point to distinct PPS or laser pulse signals.

To evaluate the timestamping errors inherent in the TRTS and LPTS methods, we implemented both approaches within our custom-developed LiDAR system for comparative analysis. An independent third-party time reference (TPTR) was integrated into the LiDAR system to accurately timestamp both PPS signals and laser pulses. The TPTR pulses exhibited a higher frequency (10 times that of TRTS or LPTS, i.e., $10^{8}$ Hz) to minimize timestamping errors. The experimental setup’s underlying principle for evaluating the timestamping errors of the TRTS and LPTS methods is illustrated in Figure 5.

The fundamental principle for evaluating the timestamping errors of TRTS and LPTS methods using TPTR is as follows: TPTR employs the same approach as TRTS to timestamp laser pulses, POS data, and laser scanner data, and then performs interpolation on the POS and laser scanner data to obtain TPTR timestamps matched with the laser pulse. Subsequently, the interpolated POS and laser scanner data corresponding to the same laser pulse are converted into TPTR timestamps under both TRTS and LPTS timestamping methods. The timestamping errors for POS data under TRTS or LPTS are then calculated by dividing the difference between the directly interpolated POS data timestamps under TPTR and the converted interpolated POS data timestamps under TRTS or LPTS by 10. The same procedure is applied to determine the timestamping errors for laser scanner data under both TRTS and LPTS methods.

The timestamping errors of TRTS and LPTS are quantified with respect to the reference TPTR method (as the higher-accuracy benchmark), facilitating comparative evaluation of their timing precision under controlled experimental conditions.

#### 4.1.1. Timestamping and Interpolating Under TPTR

The TPTR was represented by a 32-bit timer within the MCU, incrementing at a $10^{-8}$ second interval and resetting with every PPS pulse. This PPS pulse originated from a Real-Time Clock (RTC) distinct from the POS unit’s PPS for TRTS. We are assuming that each $PPS_{POS}\left(i\right)$ in (1)–(3), as well as LP(x) in (9)–(11), falls between $PPS_{RTC}\left(p\right)$ and $PPS_{RTC}\left(p+1\right)$ in Figure 5, respectively. The timestamps corresponding to the first laser pulse, the first POS data, and the first laser scanner data following $PPS_{RTC}\left(p\right)$ are TPTR(r), TPTR(s), and TPTR(t), respectively. It should be noted that the relative magnitudes of *q*, *r*, *s*, and *t* are random, and their temporal sequence in Figure 1a is for illustrative purposes only.

Then, the *x*-th laser pulse, the *y*-th POS data, and the *z*-th laser scanner data between $PPS_{RTC}\left(p\right)$ and $PPS_{RTC}\left(p+1\right)$ can be represented by (17), (18), and (19), respectively.

(17) $$\begin{array}{c} TS_{laser}^{TPTR}\left(x\right)=PPS_{RTC}\left(p\right)+TPTR\left(\frac{f_{TPTR}}{f_{laser}}\left(x-1\right)+r\pm 1\right) \end{array}$$

(18) $$\begin{array}{c} TS_{POS}^{TPTR}\left(y\right)=PPS_{RTC}\left(p\right)+TPTR\left(\frac{f_{TPTR}}{f_{POS}}\left(y-1\right)+s\pm 1\right) \end{array}$$

(19) $$\begin{array}{c} TS_{scanner}^{TPTR}\left(z\right)=PPS_{RTC}\left(p\right)+TPTR\left(\frac{f_{TPTR}}{f_{scanner}}\left(z-1\right)+t\pm 1\right) \end{array}$$

Then, the $y^{\prime \prime \prime }$-th interpolated POS data and the $z^{\prime \prime \prime }$-th interpolated laser scanner data between $TS_{POS}^{TPTR}\left(y\right)$, $TS_{POS}^{TPTR}\left(y+1\right)$ and $TS_{scanner}^{TPTR}\left(z\right)$, $TS_{scanner}^{TPTR}\left(z+1\right)$ can be represented by (20) and (21), respectively, where $y^{\prime \prime \prime }$ and $z^{\prime \prime \prime }$ are natural numbers greater than 0.

(20) $$TS_{POS}^{TPTR}\left(y^{\prime \prime \prime }\right)=TS_{POS}^{TPTR}\left(y\right)+TPTR\left(y^{\prime \prime \prime }\right)$$

(21) $$TS_{scanner}^{TPTR}\left(z^{\prime \prime \prime }\right)=TS_{scanner}^{TPTR}\left(z\right)+TPTR\left(z^{\prime \prime \prime }\right)$$

Assuming the TPTR timestamp $TS_{POS}^{TPTR}\left(y^{\prime \prime \prime }\right)$ of interpolated POS data and $TS_{scanner}^{TPTR}\left(z^{\prime \prime \prime }\right)$ of interpolated laser scanner data are equal to that of the *x*-th laser pulse, then the $y^{\prime \prime \prime }$-th interpolated POS data and $z^{\prime \prime \prime }$-th interpolated laser scanner data can be integrated with the *x*-th laser pulse to represent the *x*-th point cloud data.

#### 4.1.2. Converting Interpolated TRTS Timestamp to TPTR

As shown in Figure 5, the timestamp corresponding to the first PPS (POS) following $PPS_{RTC}\left(p\right)$ is TPTR(q). The *i*-th PPS (POS) between $PPS_{RTC}\left(p\right)$ and $PPS_{RTC}\left(p+1\right)$ can be represented by (22). It should be noted that there is only one PPS (POS) between $PPS_{RTC}\left(p\right)$ and $PPS_{RTC}\left(p+1\right)$.

(22) $$TS_{PPS(POS)}^{TPTR}\left(i\right)=PPS_{RTC}\left(p\right)+TPTR\left(q\pm 1\right)$$

Then, the $y^{\prime }$-th POS data in (4) and the $z^{\prime }$-th laser scanner data in (4) matching the *x*-th laser pulse under the TRTS method can be represented by (23) and (24) under the TPTR method, respectively.

(23) $$\begin{array}{c} TS_{POS}^{TRTS2TPTR}\left(y^{\prime }\right)=PPS_{RTC}\left(p\right)+TPTR\left(\frac{f_{TPTR}}{f_{TRTS}}TS_{POS}^{TRTS}\left(y^{\prime }\right)+q\pm 1\right) \end{array}$$

(24) $$\begin{array}{c} TS_{scanner}^{TRTS2TPTR}\left(z^{\prime }\right)=PPS_{RTC}\left(p\right)+TPTR\left(\frac{f_{TPTR}}{f_{TRTS}}TS_{scanner}^{TRTS}\left(z^{\prime }\right)+q\pm 1\right) \end{array}$$

where $f_{TPTR}÷f_{TRTS}$ is equal to 10, since the frequency of TPTR is 10 times higher than that of TRTS.

#### 4.1.3. Converting Interpolated LPTS Timestamp to TPTR

As in the previous section, the $y^{\prime \prime }$-th POS data in (12) and the $z^{\prime \prime }$-th laser scanner data in (13) matching the *x*-th laser pulse under the LPTS method can be represented by (25) and (26) under TPTR method, respectively.

(25) $$\begin{array}{c} TS_{POS}^{LPTS2TPTR}\left(y^{\prime \prime }\right)=PPS_{RTC}\left(p\right)+TPTR\left(\frac{f_{TPTR}}{f_{LPTS}}TS_{POS}^{LPTS}\left(y^{\prime \prime }\right)+r\pm 1\right) \end{array}$$

(26) $$\begin{array}{c} TS_{scanner}^{LPTS2TPTR}\left(z^{\prime \prime }\right)=PPS_{RTC}\left(p\right)+TPTR\left(\frac{f_{TPTR}}{f_{LPTS}}TS_{scanner}^{LPTS}\left(z^{\prime \prime }\right)+r\pm 1\right) \end{array}$$

where $f_{TPTR}÷f_{LPTS}$ is equal to 10, since the frequency of TPTR is 10 times higher than that of LPTS.

#### 4.1.4. TRTS and LPTS Timestamping Error Evaluation

In Figure 5, for the *x*-th laser pulse, we derived three distinct sets of interpolated POS data and laser scanner data using (20), (21), and (23)–(26), all converted to unified high-precision TPTR timestamps. Subsequently, the relative timestamping errors of both POS data and laser scanner data under the TRTS and LPTS methods were calculated separately via (27)–(30).

(27) $$\begin{array}{c} RE_{POS}^{TRTS}=\frac{f_{TRTS}}{f_{TPTR}}\left(TS_{POS}^{TRTS2TPTR}\left(y^{\prime }\right)\right.\left.-TS_{POS}^{TPTR}\left(y^{\prime \prime \prime }\right)\right) \end{array}$$

(28) $$\begin{array}{c} RE_{scanner}^{TRTS}=\frac{f_{TRTS}}{f_{TPTR}}\left(TS_{scanner}^{TRTS2TPTR}\left(z^{\prime }\right)\right.\left.-TS_{scanner}^{TPTR}\left(z^{\prime \prime \prime }\right)\right) \end{array}$$

(29) $$\begin{array}{c} RE_{POS}^{LPTS}=\frac{f_{LPTS}}{f_{TPTR}}\left(TS_{POS}^{LPTS2TPTR}\left(y^{\prime \prime }\right)\right.\left.-TS_{POS}^{TPTR}\left(y^{\prime \prime \prime }\right)\right) \end{array}$$

(30) $$\begin{array}{c} RE_{scanner}^{LPTS}=\frac{f_{LPTS}}{f_{TPTR}}\left(TS_{scanner}^{LPTS2TPTR}\left(z^{\prime \prime }\right)\right.\left.-TS_{scanner}^{TPTR}\left(z^{\prime \prime \prime }\right)\right) \end{array}$$

where $f_{TRTS}÷f_{TPTR}$ and $f_{LPTS}÷f_{TPTR}$ are equal to 0.1.

**Figure 6:**
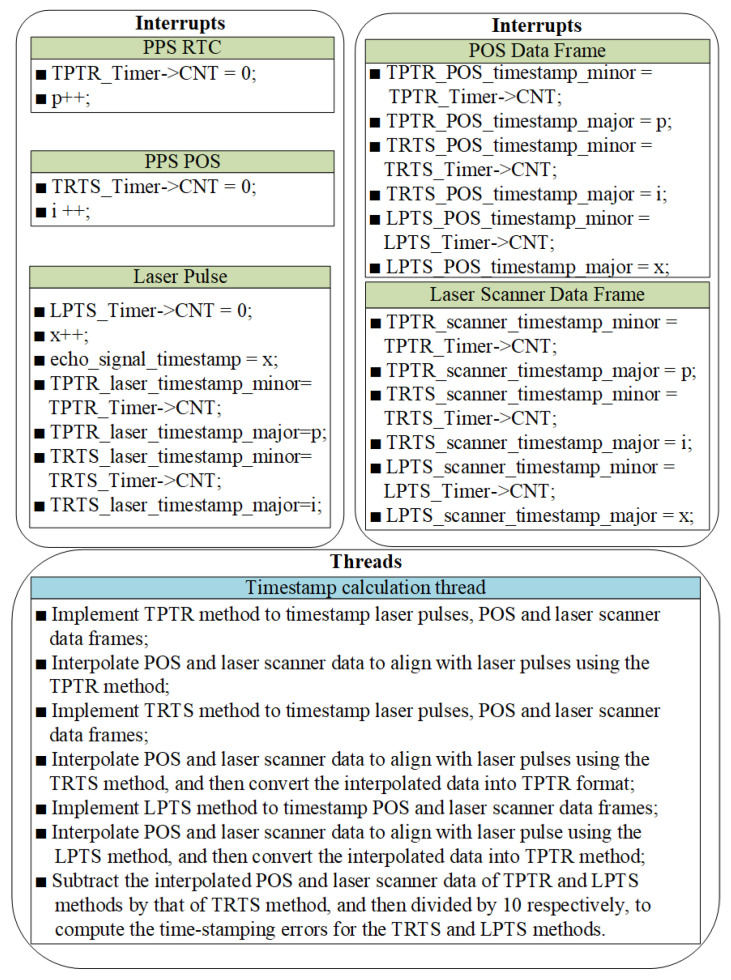
Software flowchart for timestamping error calculation.

To implement the aforementioned timestamping error evaluation framework into our custom-developed LiDAR system, we developed dedicated embedded software running on the MCU. The flowchart of the error evaluation software is presented in Figure 6. We incorporated two new interrupts into the embedded software and revised three existing interrupts. Additionally, we induced an extra thread that elucidates the specifics of the timestamping error evaluation process.

### 4.2. Results and Analysis

Building upon the principles and algorithms developed for LiDAR time synchronization, we implemented a timestamping error evaluation framework within the MCU. This implementation enabled quantitative assessment of timestamping errors between interpolated POS data, laser scanner data, and laser pulse in our custom LiDAR system, evaluating both TRTS and LPTS methods.

To validate the timestamping error evaluation framework, we conducted a series of experiments using our custom-developed LiDAR system. The experimental setup is shown in Figure 3.

The experimental process is as follows: The UAV platform was launched, and the LiDAR system was powered on. The laser unit emitted laser pulses at a rate of 2 kHz, while the POS unit provided real-time positioning and attitude data. The laser scanner unit scanned the target object, and the data acquisition and storage unit captured the full-waveform echo signal. The control unit of the custom-developed LiDAR system received the laser pulse signals from the laser unit via General Purpose Input/Output (GPIO), while acquiring data from both the POS unit and laser scanner unit through UART. The processed data were subsequently stored in the data acquisition and storage unit via an Ethernet interface, as illustrated in Figure 2.

During the experimental process, the evaluation process of TRTS and LPTS timestamping accuracy was carried out simultaneously. The timestamps of laser pulse, POS data, and laser scanner data were captured by the MCU’s embedded software through the interrupt response mechanism illustrated in Figure 6. These timestamps were subsequently processed using the computational methodology described in Section 4.1 and Figure 6 to derive the timestamping errors between the interpolated POS data and laser scanner data corresponding to identical laser pulse, with all results being recorded and stored in the data acquisition and storage unit.

**Figure 7:**
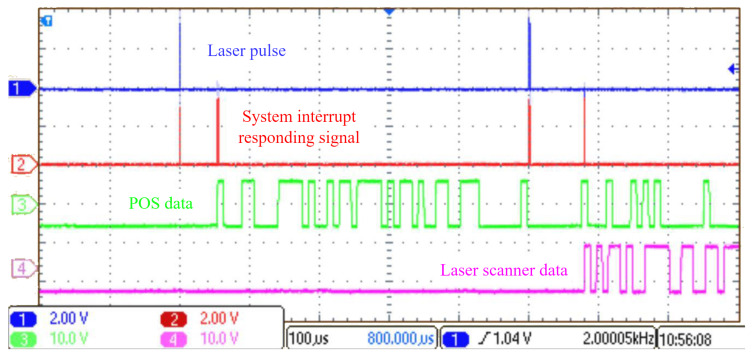
Waveforms of laser pulse (CH1), system interrupt responding signal (CH2), POS data (CH3), and laser scanner data (CH4) inside our self-developed LiDAR system are captured by oscilloscope.

For clearer demonstration of the temporal relationships between laser pulses, POS data, and laser scanner data acquired by the MCU, along with the MCU’s interrupt response to these signals, we captured the corresponding waveforms using an oscilloscope in our lab, as presented in Figure 7. The laser pulse, POS data, and laser scanner data are represented by channels CH1, CH3, and CH4, respectively. As shown in Figure 7, the micro-controller’s Interrupt Service Routine (ISR) produces precisely timed GPIO pulses coinciding with laser pulse detection, POS data reception, and laser scanner data reception onset. These pulses indicate the LiDAR system’s response to the received signals. The GPIO output waveforms are captured by CH2 of the oscilloscope.

#### 4.2.1. Timestamping Error Analysis

**Figure 8:**
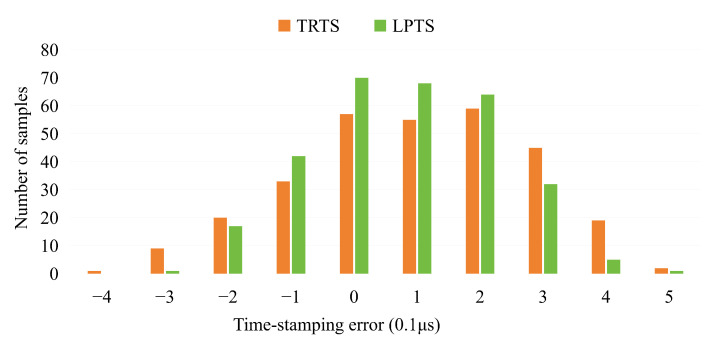
Time-stamping errors of TRTS and LPTS methods.

We have successfully obtained 300 timestamping error samples for both the TRTS and LPTS methods. Specifically, there are 200 error samples from interpolated POS data and 100 error samples from laser scanner data in both the TRTS and LPTS methods. The findings are presented in Figure 8. The horizontal axis in the figure represents the magnitude of the timestamping error, with a unit of 0.1 μs. The vertical axis represents the number of error occurrences. The orange color indicates the TRTS method, while the green color represents the LPTS method. The experimental results comparing the TRTS and LPTS methods are presented in Table 3, which provides a quantitative evaluation of their performance metrics under the same test conditions.

The better performance of the LPTS method is statistically evident in Table 3. The LPTS method achieves a higher concentration of errors than the TRTS method within ±0.1 μs (60.0% vs. 48.3%) and ±0.2 μs (87% vs. 74.7%), indicating a superior overall performance. This reduction in error variance empirically validates the theoretical model presented in Equations (8) and (16), confirming that directly driving the synchronization via laser pulses effectively eliminates the cumulative timing uncertainties inherent in third-party reference systems. These results indicate that the LPTS method achieves statistically significant reduction in synchronization error compared to TRTS, which aligns with the theoretical framework analyzing their respective time synchronization mechanisms. Both the TRTS and LPTS methods demonstrated a tendency for positive errors, potentially due to the response delay of the control unit. Beyond the central tendency, the ’tail’ analysis of the error distribution is equally revealing. Table 3 shows that, under the TRTS method, 25.3% of the samples exhibit error deviations exceeding ±0.2 μs, with a maximum observed error of 0.58 μs and a standard deviation of 0.18 μs. In contrast, under the LPTS method, only 13.0% of the samples exceed this ±0.2 μs error bound, with a maximum observed error of 0.50 μs and a standard deviation of 0.15 μs. This significant reduction in outliers suggests that LPTS is far more robust against sporadic system latencies and jitter, which is critical for reducing geometric artifacts in high-speed scanning applications. From these findings, we inferred that the LPTS method demonstrated better timestamping performance compared to the TRTS method. Additionally, system response delays might contribute to the positive bias observed in the timestamping errors of both methods.

#### 4.2.2. Worst-Case Error Analysis

While the statistical distribution of errors presented in Figure 8 highlights the general stability of the LPTS method, an analysis of the distribution tails reveals that the maximum observed absolute error for the LPTS method is 0.5 μs, compared to 0.58 μs for the TRTS method.

To evaluate the impact of these worst-case errors on point cloud quality, we performed a geometric sensitivity analysis. For a laser scanner rotating at a maximum speed of 2000 RPM (approx. ${12,000\;}^{{}^{\circ}}/s$), a timing error of 0.5 μs translates to an angular displacement of

(31) $$\Delta \theta_{max}={12000\;}^{{}^{\circ}}/s\times 0.5\times 10^{-6}\;s=0.006^{\circ }$$

Comparing this to the scanner’s mechanical encoder accuracy of $0.072^{{}^{\circ}}$, the worst-case synchronization error contributes less than 10% of the mechanical error budget. Consequently, the LPTS method does not introduce significant geometric artifacts to the LiDAR point cloud.

## 5. Conclusions

This study implements a laser pulse-driven multi-sensor time synchronization method through a custom-developed LiDAR system. We systematically analyze the timestamping precision discrepancies between LPTS and TRTS methods, with experimental validation confirming their performance characteristics. The principal conclusions derived from the experimental data are as follows:

(1) Theoretical analysis reveals that the LPTS method exhibits a timestamping error of ±1, contrasting with the TRTS method’s twofold greater error margin (±2). This conclusion aligns with experimental data in Figure 8, where LPTS demonstrates better error distribution characteristics, with timestamping error deviations predominantly clustered around zero compared to TRTS.

(2) The responding delay of the control system contributes a positive timestamping error for both the LPTS and TRTS methods.

Through integrated theoretical modeling and experimental validation, our findings demonstrate that the adoption of an event-driven architecture effectively decouples internal synchronization precision from the stability of external GNSS signals. Specifically, the LPTS method exhibits a timestamping error of ±1, enabling 0.1 μs level time synchronization accuracy in our custom-developed LiDAR system.

However, it is important to note that the achievement of high-precision time synchronization, whether using LPTS or TRTS, relies on high-precision internal timers of the MCU and the MCU’s rapid response to interrupts. To pursue even higher time synchronization accuracy, it is advisable to select an MCU with stronger real-time performance and a more precise clock source when designing the LiDAR system. Furthermore, it is imperative to enhance the response to interrupt events during the design phase of the control system software.

## Figures and Tables

**Table 1:** Advantages and disadvantages of different time synchronization methods.

Time Sync. ^1^ Method	Pros ^2^	Cons ^3^
Hardware sync.	High precision, high sync. speed, simple software	Additional hardware, complex hardware
Software sync.	No additional hardware, relatively high precision	Complex software, high computational resources
Hybrid sync.	Relatively high precision, relatively high sync. speed	Additional hardware, simple hardware

^1^ Sync.: Synchronization; ^2^ Pros: Advantages; ^3^ Cons: Disadvantages.

**Table 2:** Key performances of our custom-developed LiDAR system.

Parameters	Value
Carrier platform	UAV
Payload capacity (kg)	25
Flight altitude (m)	50
Flight speed (m/s)	20
Weight of LiDAR (kg)	18
Electrical power (W)	200
Laser wavelength (nm)	532
Laser pulse width (ns)	3
Laser repeated rate (Hz)	2000
Scanning angle (deg)	±10
POS data rate (Hz)	100

**Table 3:** Comparative table of experimental results between the TRTS and LPTS methods.

Properties	TRTS	LPTS
PPS source needed?	Yes	No
Laser jitter avoided?	No	Yes
Theoretical timestamping error	±2	±1
Experimental error within ±0.1 μs	48.3%	60.0%
Experimental error within ±0.2 μs	74.7%	87.0%
Maximum observed error	0.58 μs	0.50 μs
Standard deviation	0.18 μs	0.15 μs

## Data Availability

The original contributions presented in this study are included in the article. Further inquiries can be directed to the corresponding author.
